# Exploring Gut Microbiota and the Influence of Physical Activity Interventions on Overweight and Obese Children and Adolescents: A Systematic Review

**DOI:** 10.3390/healthcare11172459

**Published:** 2023-09-03

**Authors:** Micaela C. Morgado, Mónica Sousa, André B. Coelho, Júlio A. Costa, André Seabra

**Affiliations:** 1 Research Centre in Physical Activity, Health and Leisure (CIAFEL), Faculty of Sport, University of Porto, 4200-450 Porto, Portugal; andre.seabra@fpf.pt; 2 Portugal Football School, Portuguese Football Federation (FPF), 1495-433 Cruz Quebrada, Portugal; jahdc@hotmail.com; 3 CIDEFES, Universidade Lusófona, 1749-024 Lisboa, Portugal; 4 CINTESIS@RISE, NOVA Medical School (NMS), Faculdade de Ciências Médicas (FCM), Universidade Nova de Lisboa, 1169-056 Lisboa, Portugal; 5 Faculty of Sports Science and Physical Education, University of Coimbra, 3040-248 Coimbra, Portugal; andrebastoscoelho@hotmail.com

**Keywords:** childhood, obesity, lifestyle, exercise, intestinal microbiota, *Bacteroidetes*, microbiota diversity

## Abstract

The recognition that the gut microbiota of obese children differs from lean children has grown, and some studies suggest that physical activity positively influences the gut microbiota. This systematic review explores the changes in the gut microbiota composition of obese and non-obese children and adolescents and provides an understanding of the effects of physical activity interventions in modulating their microbiota. The PRISMA protocol was used across PubMed, Scopus, and Web of Science. Overall, twenty-four research papers were included in accordance with the chosen inclusion and exclusion criteria, eighteen studies compared the gut microbiota of obese and normal-weight children and adolescents, and six studies explored the effect of physical activity interventions on the gut microbiota. The analysis indicated that obese gut microbiota is reduced in *Bacteroidetes*, *Bifidobacterium* and alpha diversity but enriched in *Proteobacteria* and *Lactobacillus*. Interventions with physical activity seem to improve the alpha diversity and beneficial bacteria linked to body weight loss in children and adolescents. The gut microbiota of obese children exhibited a remarkably individual variation. More interventions are needed to clearly and accurately explore the relationships between child obesity, gut microbiota, and physical activity and to develop approaches to decrease the incidence of paediatric obesity.

## 1. Introduction

The global prevalence of childhood overweight and obesity has reached epidemic magnitudes. In 2016, the number of overweight or obese children and adolescents between the ages of 5 and 19 exceeded 340 million worldwide [1]. Between 1975 and 2016, the world prevalence of overweight and obesity among children and adolescents aged 5 to 19 has undergone a significant escalation, rising dramatically from a mere 4% to slightly above 18% in both boys and girls [1]. In the WHO European Region, for children aged 5 to 9 years old, there is a notable rise in the prevalence of overweight and obesity, with approximately one in eight children classified as obese and nearly one in three classifieds as overweight [2]. 

The aetiology of childhood obesity is widely acknowledged to encompass an interplay of factors, including genetic predisposition and environmental influences [3]. Furthermore, children and adolescents with overweight or obesity have an increased likelihood of experiencing obesity and its consequential health complications into adulthood [4]. So, prioritizing children’s health through interventions that target modifiable risk factors, such as physical activity and dietary habits, has been a focal point of prevention strategies, holding the potential to address and solve this health issue [5].

In the last years, an emerging area of intervention for modifiable risk factors has centred around the gut microbiota, a complex of microorganisms that live collectively in the gastrointestinal tract [6]. The gut microbiota is known as a significant factor that establishes connections between genetic factors, external factors, and the immune system [6], developing a mutually beneficial relationship with the host [7,8] and playing a crucial role in regulating human metabolic balance [9].

The literature suggests that the colonization of the gut microbiota may be initiated during prenatal development [10]. Then, its diversity starts developing with birth, and the mode of delivery plays a crucial role in its formation during the first years of life [11]. Exogenous factors can influence the progression of this colonization process, leading to the transition to a gut microbiota similar to that of an adult, such as methods of milk feeding (breastfeeding or artificial milk), the introduction of solid foods, diet and cultural habits, diseases, and exposure to antibiotics [11,12,13]. If the microbial ecosystem is disturbed, the mutualistic relationship between the microbial communities and the host may break down—this change is called dysbiosis [13]. There is compelling evidence connecting the dysbiosis of the gut microbiota with the pathophysiology of obesity [9] and other metabolic disorders [14]. Particularly in childhood, the gut microbiota composition has been recognized as a potential determinant of obesity since this period of life is pivotal for the establishment of gut bacteria colonization [15]. Research suggests that an elevated *Firmicutes* to *Bacteroidetes* ratio may be associated with obesity and metabolic disturbances in children [15]. Earlier investigations have highlighted that the diversity and richness of gut microbiota in obese children substantially lag those found in their normal weight counterparts [16].

The level of body mass index, frequency of exercise, dietary habits and cultural practices have been the subject of study and have been revealed to influence the infant microbiota [13,17,18,19], contributing to the normal balance [20]. Specifically, exercise is acknowledged for stabilizing obesity development and modifying the gut microbiota composition, leading to increased bacterial diversity [19,21,22]. On the other hand, exercise also seems to modulate the *Firmicutes* to *Bacteroidetes* ratio, which can be related to body weight reduction [22], reducing body fat and enhancing the bacterial diversity of the gut microbiota [23,24]. Exercise promotion may help to preserve the composition of the gut microbiota or re-establish the normal balance, promoting eubiosis [25]. For these reasons, considerable interest has focused on modifiable factors, namely, physical activity and a balanced diet [12,26,27,28]. Consequently, the manipulation of the gut microbiota holds promise as a potential therapeutic avenue for addressing metabolic disorders among the paediatric population [6,29,30,31,32].

Therefore, the present study constitutes a systematic literature review focusing on two different and complemented searches. Firstly, the primary aim is to investigate and describe the profiles of gut microbiota in both obese and normal weight children and adolescents. Secondly, this study intends to provide insights into the effectiveness of physical activity interventions in shaping the gut microbiota composition among overweight and obese children and adolescents. This systematic review aims to open novel avenues for the prevention of childhood obesity.

## 2. Materials and Methods

This systematic review was carried out following the guidelines of the Preferred Reporting Items for Systematic Reviews and Meta-Analyses (PRISMA) 2020 [33]. Given the scope of the current systematic review in physical activity, the implementation of PRISMA in Exercise, Rehabilitation, Sports medicine, and SporTs science (PERSiST) guidance was also adopted [34]. The systematic review protocol was registered at the International Platform of Registered Systematic Review and Meta-analysis Protocols (INPLASY202370045).

### 2.1. Search Strategy

A systematic literature search was conducted in three databases: PubMed, Scopus, and Web of Science. The search was conducted on articles published within the last decade, spanning from 2010 to June 2023. This period marked a phase during which research on this topic gained heightened prominence. To increase the efficiency of the search and obtain all synonyms gathered in a single descriptor, the search was performed using single-text words in the title and abstract, complemented with Medical Subject Headings (MeSH) terms.

First search: type of population (“Child*” OR “Paediatric*” OR “Infant*”) AND health condition (“Obes*” OR “Overweight”) AND outcome of interest (“Gut Microbio*” OR “Microbio*”).

Second search: type of intervention (“Physical Activity” OR “Exercise”) AND type of population (“Child*” OR “Paediatric” OR “Infant”) AND health condition (“Obes*” OR “Overweight”) AND outcome of interest (“Gut Microbio*” OR “Microbio*”).

To identify additional relevant studies, we used the “related citations” function in PubMed and thoroughly examined the reference list of each selected article.

### 2.2. Eligibility Criteria

The eligibility criteria were framed in accordance with the PICO framework in accordance with the PRISMA statement: population, intervention, comparison, and outcomes [35]. Participants and setting: children and adolescents with overweight and obesity [36]; Interventions: physical activity and lifestyle programs; Control: normal weight children and adolescents; Outcomes: changes and differences in the composition of the gut microbiota.

The following inclusion criteria were applied: articles in English, studies involving children and adolescents, studies comparing the composition of the gut microbiota in children and adolescents with and without obesity, and studies evaluating physical activity interventions on the composition of the gut microbiota in children and adolescents. The following exclusion criteria were adopted: children and adolescents with other disease(s), subjects aged under 3 and over 18 years, animal model studies (e.g., mice, rats, pigs, or in vitro), studies with microbiota from other organs (e.g., mouth, nose, skin, or vagina), studies with drugs or supplements (e.g., antibiotic, prebiotic, or probiotic), and editorials, reviews, or meta-analyses.

### 2.3. Study Selection

Reviewers extracted all identified records using a standardized data extracting form (Mendeley Reference Manager 2.94.0, Elsevier, London, United Kingdom), and duplicate publications of the same study were excluded using the “duplicates” function of Mendeley Reference Manager and manually removed after manual check by M.M. and A.C. The search process and study reviews were conducted independently by two authors (M.M. and A.C.). In the event of ambiguity in the title, the abstract was checked for verification using the eligibility criteria. In case of any doubt about inclusion in the study or disagreement, inclusion was debated with A.S. and M.S., and it was decided by consensus between the reviewers. Full-text articles attending to the mentioned criteria were downloaded, archived, and then read and evaluated by the reviewers for the systematic review. 

### 2.4. Data Extraction

A thorough screening of the selected articles was conducted to extract the relevant information regarding the study identification (i.e., authors and year of publication), the characteristics of the participants (i.e., number of participants, age, and BMI), the location where the study was carried out, the study design, the methodology, the type and duration of the intervention; the synthesis of the main findings was achieved by one author (M.M.), and successively reviewed by other authors (A.S. and M.S.). In the synthesis of main findings, these outcomes were described if available: differences in the *Firmicutes* to *Bacteroidetes* ratio, differences in the bacterial composition of the gut microbiota, and differences in the alpha- and beta-diversity.

### 2.5. Risk of Bias

The assessment of study biases was carried out using the Cochrane tools. Specifically, the Risk of Bias in Non-randomized Studies of Exposures (ROBINS-E) tool was employed for the first search [37], while for the second search, the Risk of Bias in Non-randomized Studies of Interventions (ROBINS-I) tool was used [38]. ROBINS-E encompasses seven bias domains, with a judgment scale consisting of “low risk”, “some concerns”, “high risk”, and “very high risk” of bias [37]. Similarly, ROBINS-I addresses seven distinct bias domains, categorizing bias risk assessments as “Low risk”, “Moderate risk”, “Serious risk”, and “Critical risk” [38].

## 3. Results

### 3.1. Study Selection and Study Characteristics

#### 3.1.1. Gut Microbiota of Children with Overweight or Obesity

**Figure 1:**
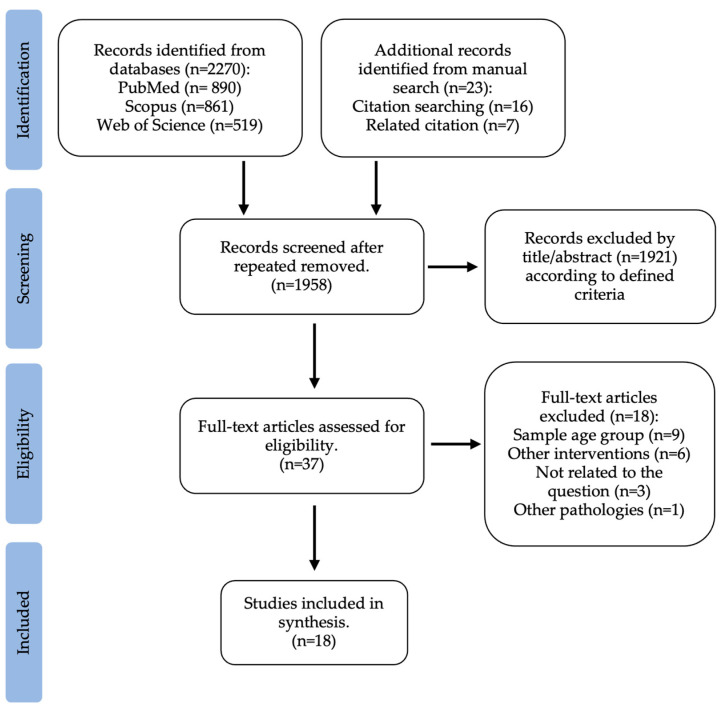
Flow diagram of study selection: gut microbiota of obese and normal weight children.

The first search carried out was on the gut microbiota of overweight or obese children in comparison to the gut microbiota of normal weight children, according to previously defined criteria. The literature search identified a total of 2270 potentially relevant articles (PubMed: 890; Scopus: 861; Web of Science: 519). This search was supplemented with 23 studies from “related citations” and a manual search of reference lists. After removing repeated articles, 1958 were considered for assessment. From this, 1921 were excluded by title or abstract. This way, 37 articles were selected for full-text review. Ten were not related to the question, and nine were excluded because the age group did not match the research. An overall of 18 studies that fulfilled the inclusion criteria were considered in this systematic review for qualitative analysis. The selection process of the included studies is summarized in Figure 1.

The selected articles were published from 2012 to 2022, were cross-sectional studies, and were conducted in children and adolescents between 3 and 18 years old from various ethnicities: nine were Asian [16,39,40,41,42,43,44,45,46], four were Latino [47,48,49,50], four were Caucasian [51,52,53,54] and one was mixed [55]. The methods of faecal microbiota analysis were mostly 16S rRNA gene sequencing, one study used Shotgun metagenomics [47], and another one used quantitative real-time PCR (qPCR) analysis [45]. All the studies search for inter-individual variations according to body mass index level.

#### 3.1.2. Physical Activity Impact in Children’ and Adolescents Gut Microbiota

**Figure 2:**
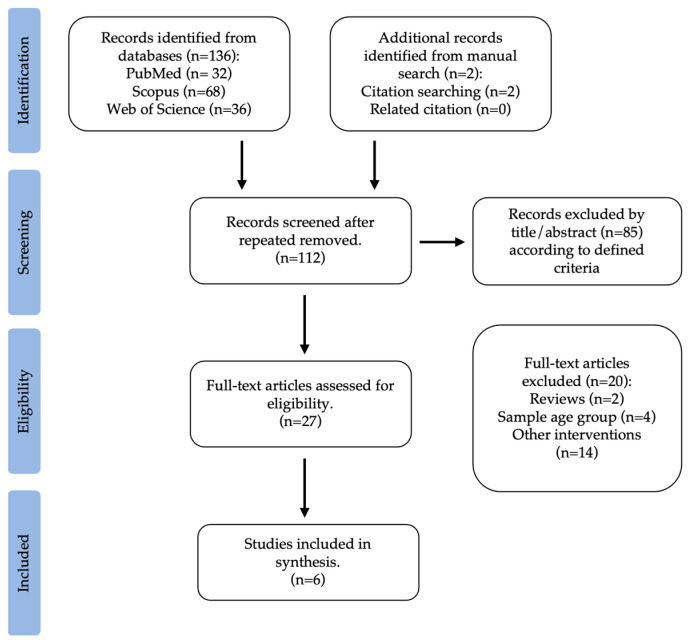
Flow diagram of study selection: physical activity interventions and the gut microbiota in children and adolescents with overweight and obesity.

The second search carried out was on interventions with physical activity and their impact on the gut microbiota of overweight and obese children and adolescents, according to previously defined criteria. The literature search provided a total of 136 original citations (PubMed: 32; Scopus: 68; Web of Science: 36). This search was supplemented with two studies from a manual search of reference lists. After removing repeated articles, 112 were considered for assessment. From this, 85 were excluded by title and abstract. This way, 27 articles, chosen based on the title and abstract, underwent a comprehensive full-text review. Two were reviews, four were excluded because the age group did not match the research, and fifteen were related to other questions (e.g., environment, diet, preconception, geography, or diseases). Lastly, an overall of six studies were selected, according to previously defined reasons, whose objective was to explore the connection between childhood obesity, physical activity, and gut microbiota. Figure 2 illustrates the selection process for this systematic review.

The selected articles were published from 2009 to 2022; five were intervention longitudinal studies [19,23,56,57,58], and one was retrospective [59]. Studies were conducted on children between 7 and 18 years old from the following ethnicities: Latino [56], Asian [19,57], Caucasian [23,58], and mixed [59]. The methods of faecal microbiota analysis were mostly 16S rRNA gene sequencing, and one used quantitative real-time PCR (qPCR) analysis [58]. The duration of the interventions ranged from 6 weeks to 12 weeks; two studies consisted of physical activity recommendations [19,56], three interventions prescribed a detailed exercise program [23,57,58], and one study applied a retrospective questionnaire about exercise frequency [59]. Furthermore, four interventions had simultaneously nutritional counselling [19,56,57,58]. Despite the different intervention approaches, given the scarcity of articles on this topic, we considered six articles for detailed analysis. 

### 3.2. Risk of Bias

**Figure 3:**
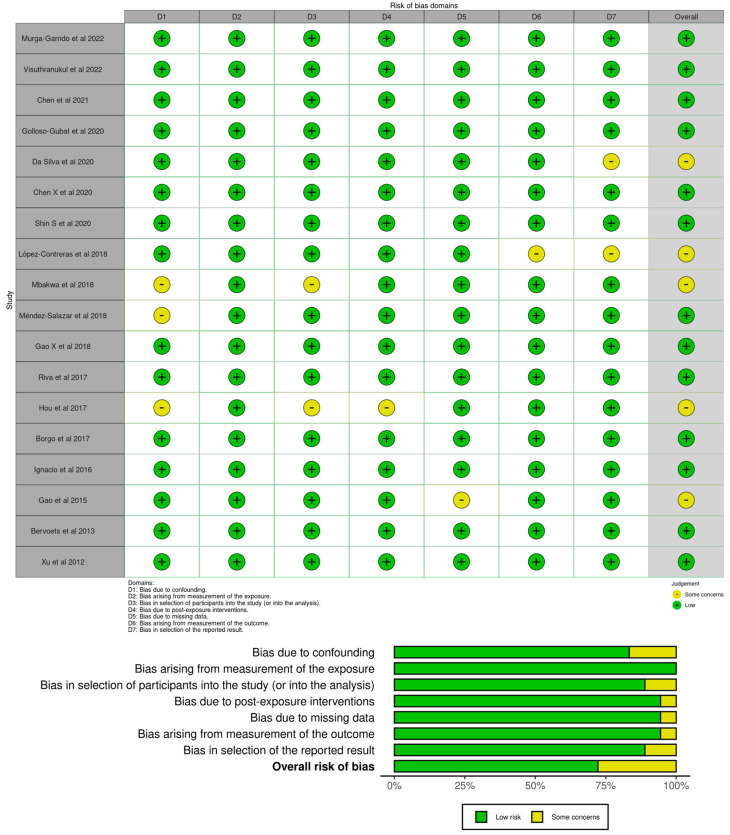
Risk of bias judgments for the gut microbiota profiles in children with obese and normal weight through ROBINS-E [16,39,40,41,42,43,44,45,46,47,48,49,50,51,52,53,54,55].

For the first search, the “measurement of exposure” was judged as low risk in 100% of the studies. For most of the studies (*n* = 17) the “post-exposure interventions”, “missing data”, and the “measurement of the outcome” domains were judged as having a low risk of selection of bias (94.4%), with one study being judged as having some concerns. For most of the studies (*n* = 16), the “selection of participants into the study (or into the analysis)” and the “selection of the reported result” domains were judged as low risk of selection of bias (88.9%), with two studies being judged as some concerns. For most of the studies (*n* = 15), the “confounding” domain was judged as having a low risk of selection of bias (83.3%), with three studies being judged as having some concerns. A total of thirteen studies were judged as low risk in the overall risk of bias (72.2%) and five studies as some concerns (27.8%). No studies were judged with high risk or very high risk of bias for each domain. Figure 3 illustrates the robvis tool [60] risk of bias assessment for the articles selected in the first search.

**Figure 4:**
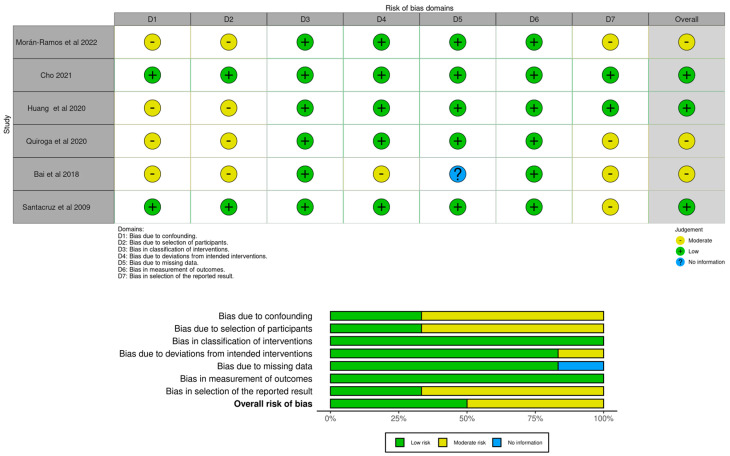
Risk of bias judgments for effectiveness of physical activity interventions in gut microbiota modulation in overweight or obese children and adolescents through ROBINS-I [19,23,56,57,58,59].

For the second search, the “classification of interventions” and the “measurement of outcomes” were judged as low risk in 100% of the studies. For most of the studies (*n* = 5), the “deviations from intended interventions” and the “missing data” domains were judged as low risk of selection of bias (83.3%), with one study being judged as moderate risk and the other as no information. For 66.7% of the studies (*n* = 4), the “confounding”, “selection of participants” and “selection of the reported result” domains were judged as moderate risk, with two studies being judged as low risk. Regarding the overall risk of bias, three studies were judged as low risk (50%) and another three studies as moderate risk (50%). No studies were judged with either serious or critical risk of bias for each domain. Figure 4 illustrates the robvis tool [60] risk of bias assessment for the articles selected in the second search.

### 3.3. Gut Microbiota Profiles in Overweight and Obese Children

Regarding *Firmicutes*, two studies reported that the gut microbiota of children with overweight or obesity was characterized by a higher abundance of *Firmicutes* compared to normal weight [52,55], while another one reported the opposite [43]. With respect to phylum *Bacteroidetes*, major studies related that children with overweight and obesity had lower proportions of *Bacteroidetes* than children with normal weight [16,39,40,42,49,52], while one study observed significantly higher proportions in the obese children [43]. Concerning the Actinobacteria phylum, three studies observed a decrease in the relative abundance in children with overweight and obesity [39,40,43]. The *Proteobacteria phylum* relative abundance was higher in the obese children [39,40,49], as well as Fusobacteria [39,40], while the relative abundances of *Verrucomicrobia* were lower in the obesity group [43,44]. In contrast, another study reported no significant variations in the phyla relative abundance between obese and normal weight children [48].

Regarding the *Firmicutes* to *Bacteroidetes* ratio, four studies reported a significant increase among obese children when compared to children with normal weight [42,44,52,54]. In contrast, one study found a significantly lower *Bacteroidetes* to *Firmicutes* ratio in children from the obesity group compared to both the normal weight and overweight groups [46].

Concerning genera level, studies have reported a higher relative abundance of *Lactobacillus* in children with overweight or obesity compared to those of normal weight [39,55], as well as *Faecalibacterium* [16], *Lachnospira* [16,41], *Prevotella* [40], *Actinomyces*, *Romboutsia*, *Weissella* [42], *Blautia* [39,44], *Enterococcus*, *Sutterella*, *Klebsiella,* and *Collinsella* in the group of obese children [44]. Bacteroides levels were found to be significantly lower in the children from the obesity group than in children from the normal weight group [46], as well as *Bifidobacterium* [39,41,45,55], *Oscillospira,* and *Dialister* [16].

The most abundant species of bacteria detected in obese children was *Bacteroides plebeius*, *Bacteroides dorei*, *Bilophila wadsworthia*, *Clostridium symbiosum*, *Parabacteroides distasonis*, *Parasutterella excrementihominis* [43], *Bacteroides eggerthii* [48], *Bacteroides fragilis* [50], *Eubacterium* sp. and *Roseburia* sp. [47], *E. coli* [45] and *Lactobacillus* spp. compared to non-obese children [50,54]. Instead, overweight and obese children showed a lower relative abundance of *Akkermansia muciniphila* [53], *Bifidobacterium* spp. [50], *Bacteroides vulgatus* [54], *Candida* spp., *Faecalibacterium prausnitzii* and *Saccharomyces* spp. than non-obese children [53].

Concerning alpha diversity, overweight and obese children presented a significant reduction in comparison to non-obese individuals across the five studies [16,40,43,49,55], whereas another five articles found no differences [39,41,42,47,48]. When analysing beta diversity, some authors noted significant differences between groups [42], while others found no differences [41].

Some positive correlations between bacteria and body weight were described by the authors, such as a positive correlation between the relative abundance of *Firmicutes* [55], *Bacteroides fragilis* [50], *Lactobacillus* spp. [50], and BMI; the relative abundance of the *Lachnospiraceae* family [42], and *Firmicutes phylum* [52] was positively correlated with BMI z-score; the relative abundance of *Bacteroides eggerthii* exhibited a positive correlation with BMI percentile and percentage of body fat [48].

Nevertheless, studies also reported negative correlations; three reviewed studies reported a negative correlation between *Bacteroidetes phylum* and BMI z-score in children [42,46,52], namely with the relative abundance of the *Bacteroidaceae* family [42]. The *Bifidobacterium* spp. was negatively correlated with BMI [50] and *Akkermansia* showed an inverse association with BMI z-score, weight z-scores and overweight [51].

An overview of the studies included in this systematic review is presented in Table 1.

### 3.4. Physical Activity Interventions on Gut Microbiota in Children and Adolescents with Overweight and Obesity

A great diversity of results was observed between studies. For instance, in a 6-week multidimensional intervention with physical activation recommendations to achieve at least 150 min/week and nutritional education, the composition and diversity of the gut microbiota of the obese participants remained unchanged [56]. However, in another 6-week intervention with obese adolescents, performed a moderate- and high-intensity exercise program, which included endurance and strength training over 6 weeks (5 h per day for 6 days a week) and a calorie-restricted diet, authors observed a significantly higher alpha diversity and species richness after intervention [57]. Moreover, research that investigated associations between exercise frequency through a self-reported questionnaire and gut microbiota found a significant correlation between exercise frequency and alpha diversity [59]. Furthermore, in a weight reduction program with individual nutritional and physical activity counselling with obese Korean children, after 8 weeks the bacterial richness was lower in the group with higher fat loss, with no differences in beta diversity [19].

Concerning the phylum level, some authors found higher levels in the relative abundance of *Firmicutes* [19,59], while others observed the opposite after the intervention [57]. The same was reported regarding the *Bacteroidetes phylum*, which decreased after an 8-week weight reduction program [19] but increased after intense exercise training [57]. Moreover, the intense exercise training resulted in a reduction in the *Firmicutes* to *Bactoroidetes* ratio after the intervention [57]. The Actinobacteria phylum relative abundance was significantly reduced in relation to physical activity frequency [59]. Moreover, the *Proteobacteria phylum* decreased after a training program [23].

With respect to the genus level, studies reported a decrease in the *Bacteroides* genus [19] and *Streptococcus* [57], while *Lactobacillus* [58], *Roseburia*, *Blautia and Dialister* increased after intervention [23]. These three bacterial genera were observed to have a lower relative abundance before the intervention in children with obesity, making it similar to the profile of healthy children after the intervention [23].

Regarding the species level, only one study reported changes after the intervention; specifically, participants showed an increase in *Bacteroides fragilis*, while the species *Bifidobacterium adolescents*, *Bifidobacterium longum*, and *Clostridium coccoides* decreased after a 10-week intervention based on regular physical activity and an energy-restricted diet [58]. 

**Figure 5:**
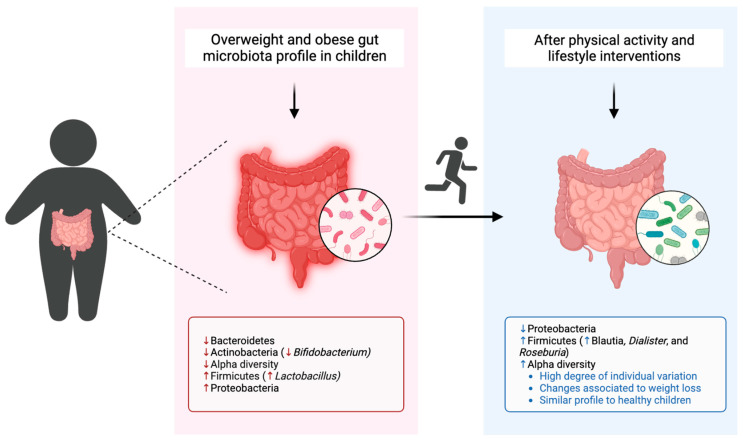
Gut microbiota composition of children with overweight and obesity in comparison to healthy children and modulation after physical activity interventions. Created with BioRender.com (https://app.biorender.com/ (accessed on 11 July 2023)).

An overview of the studies included in this systematic review is presented in Table 2, and a resume of the predominant composition of the gut microbiota in children who are overweight or obese, in comparison to those with normal weight and their gut microbiota modulation after physical activity interventions is illustrated in Figure 5.

## 4. Discussion

This systematic review has revealed that gut microbiota modulation through lifestyle interventions, such as the increase in physical activity, can be an effective method for preventing and treating childhood obesity [6]. Furthermore, some authors have proposed that the Bacteroidetes to *Firmicutes* ratio could be used as a biomarker of vulnerability for the development of obesity [30,53,54,58].

In healthy children, the gut microbiota is related to a lower *Firmicutes* to *Bacteroidetes* ratio, whereas in children with obesity, this ratio seems to be reversed, showing a greater relative abundance of *Firmicutes* and a reduced relative abundance of *Bacteroidetes* [30,40,44,52,54,55,66]. However, the findings remain contradictory. Several inconsistencies associated with the *Firmicutes* to *Bacteroidetes* ratio in obese children were reported; some authors described an increase [42,44,52,54,55], a decrease [46], or no change at all [16,39,41,47,48,49,51]. Nevertheless, it should be noted that an intense exercise program with calorie dietary restriction over 6 weeks was able to significantly reduce the *Firmicutes* to Bacteroidetes ratio while improving alpha diversity in obese adolescents [57].

Moreover, some authors reported an association between higher levels of bacteria from the *Firmicutes phylum* and overweight or obesity, in particular, *Lactobacillus* spp. [50,54,55,59,66]. In the study conducted by Bervoets et al., which examined the differences in the gut microbiota composition in obese and lean children, a positive association between *Lactobacillus* spp. and plasma high-sensitive C-reactive protein (hsCRP) levels in the obese subjects were found, suggesting that *Lactobacillus* spp. may potentially have an impact in low-grade inflammation [54]. Likewise, in another study with Egyptian children and adults, it was revealed that hsCRP levels were notably elevated in individuals with obesity, and a positive correlation was observed between higher hsCRP levels and the presence of *Firmicutes* in the subjects, such as *Lactobacillus* spp., was showed [67]. In adults with obesity, a significantly higher *Lactobacillus* spp. concentration than those without obesity was also found [68]. Of note, after a weight reduction program where adolescents received a hypocaloric diet and a physical activity prescription, the counts of the *Lactobacillus* group were significantly reduced in the adolescents who experienced the highest weight loss compared to those in the group with low weight loss [58]. The consistent results regarding *Lactobacillus* spp. highlight a robust link between the relative abundance of the *Lactobacillus* genus and obesity. These findings suggest that *Lactobacillus* spp. may play a potential role in body weight regulation and the development of obesity.

On the other hand, children with obesity seem to have an increase in *Proteobacteria* [39,40,44,49,59], but a combined training program could have the potential to decrease these bacteria, namely, the *Gammaproteobacterial* class [23]. Another interesting observation is that a reduced relative abundance of *Bifidobacterium* is often related to children with overweight and obesity in comparison to those with normal weight [41,45,50,55]. It was previously reported that differences in the composition of children’s gut microbiota may occur prior to the onset of obesity, namely, reductions associated with the *Bifidobacterium* genus [69]. In adults, a reduction in the relative abundance of *Bifidobacterium* was observed in individuals with an increased visceral fat area [70] and in individuals with obesity based on BMI criteria [29]. Moreover, the species *Bifidobacterium longum* was recently correlated with a high visceral fat area in adults [71]. The relative abundance of *Bifidobacterium* in relation to other bacterial collections is believed to hold significance in the context of obesity rather than focusing solely on their absolute numbers [58].

It is important to acknowledge that the analysis of the gut microbiota composition is susceptible to significant variations among individuals [53]. The reported high variation makes it challenging to determine the level of variability in response across different studies. Variable results could also be explained by differences in the study design, the limited number of children within a given sample, and confounding factors such as different types of physical activity interventions [72], diet [73], geographical effects and cultural traditions [74], age-range and pubertal status [75], or even different sequencing techniques [76]. Four selected studies had simultaneously physical activity and nutritional counselling [19,56,57,58]. Diet is also a factor that influences the human gut microbiota [73,77,78,79]. Thus, some changes in the gut microbiota that seem to be associated with physical activity may, therefore, be due to changes in nutritional intake [80]. Although, the interventions that combined both physical activity and nutritional counselling presented in this systematic review did not find changes associated with dietary factors and did not report differences in the nutritional intake over the interventions [19,56,57,58].

While previous reviews have explored the effects of physical activity in adults, this systematic review represents a novel endeavour in assessing and consolidating the current knowledge regarding the gut microbiota profile and the influence of physical activity interventions on the gut microbiota composition in a specific population, such as children and adolescents, who are overweight or obese. However, during our search process, we focused on studies published in English, conducting searches in PubMed, Scopus, and Web of Science. It is important to note that this restriction might have unintentionally excluded potentially valuable studies published in other languages. To gain a deeper insight into the potential impact of physical activity on gut microbiota composition, it would be advantageous that future studies include a predefined set of gut microbiota variables that have already been established as relevant, specifically on the relative abundance of bacteria such as *Firmicutes*, *Bacteroidetes*, *Actinobacteria*, *Proteobacteria*, *Lactobacillus* and *Bifidobacterium*, the metrics of the *Firmicutes* to *Bacteroidetes* ratio, as well as the values concerning to bacterial diversity. These reports will remain highly pertinent, even in cases where the outcome demonstrates no discernible change or difference.

Nevertheless, exercise training and weight loss among overweight and obese children and adolescents were discovered to act as a dispersing factor. Specifically, the bacterial communities shifted after the physical activity interventions, resulting in a gut microbiota composition similar to that of healthy counterparts [23,58]. The observed results in the examined studies reflect that gut microbiota might be an issue that, combined with a healthy lifestyle, contributes to the regulation of body weight [58]. Children have an intrinsically plastic gut microbiota with a heterogeneous population, revealing the potential for preventive and therapeutic interventions to improve children’s health [6].

## 5. Conclusions

Obesity is a complex and multifactorial disorder that interacts with the gut microbiota. Based on this systematic review, it has been observed that variations in the gut microbiota exist in relation to body weight, and both the diversity and the total amount of bacteria differ, contributing to what appears to be an obesogenic profile. Additionally, physical activity interventions revealed changes in the gut microbiota of overweight and obese children and adolescents. Despite the degree of individual variation, the literature shows that interventions with physical activity may have an impact on the development and modulation of the young gut microbiota associated with weight loss, making it similar to the profile of healthy children. Nevertheless, the real impact that physical activity can have on gut microbiota in children and adolescents continues to be studied. Studies are scarce and with different methodologies, which demonstrates the need to carry out more research in this age group, standardize protocols and carry out investigations that analyze the effect of physical activity in isolation, that control and standardize the dietary intake of participants to minimize cofounders, to identify the interplay between physical activity and gut microbiota composition in children with overweight and obesity, and draw clear conclusions. These findings should work as drivers of healthy lifestyles that promote adequate body weight at each stage of the child’s growth and the development of an equally healthy intestinal microbiota.

## Figures and Tables

**Table 1:** Gut Microbiota composition in overweight and obese children compared to normal weight.

Reference	Country	Sample	Aim	Method of Faecal Microbiota Analysis	Observed Results (Overweight/Obese vs. Normal Weight Children)
Murga-Garrido et al., 2022 [47]	Mexico	*n* = 46 Age: 6–12 years Ethnicity: Latino Obese [61]: *n* = 20 Normal weight: *n* = 26	To explore the gut microbiota characteristics, stratified based on their dietary profile and body mass index	Shotgun metagenomics	↑ *Eubacterium* sp. and *Roseburia* sp. ≠ Alpha-diversity ≠ F/B ratio
Visuthranukul et al., 2022 [39]	Thailan	*n* = 164 Age: 7–15 years Ethnicity: Asian Obese [61]: *n* = 164 Normal weight: *n* = 45	To compare the gut microbiota of obese and healthy controls and investigate associations between the microbiome, lifestyle, adiposity, and metabolic profiles	16S rRNA gene sequencing	↓ *Bacteroidetes* and *Actinobacteria* ↓ *Bifidobacterium* ↑ *Blautia* and *Lactobacillus* ↑ *Proteobacteria* and *Fusobacteria* ≠ Alpha-diversity ≠ F/B ratio
Chen et al., 2021 [40]	China	*n* = 100 Age: 6–11 years Ethnicity: Asian Overweight [62]: *n* = 35 Obese: *n* = 35 Normal weight: *n* = 30	To characterize the gut microbiota in children across different weight categories	16S rRNA gene sequencing	↓ species abundance ↓ *Bacteroidetes* ↓ *Actinobacteria* and *Tenericutes phyla* ↑ *Aggregatibacter*, *Fusobacterium*, *Haemophilus, Megamonas*, *Prevotella*, *Sneathia*, *Sutterella* and *Veillonella* ↑ *Proteobacteria* and *Fusobacteria* ≠ F/B ratio
Golloso-Gubat et al., 2020 [41]	Philippines	*n* = 43 Age: 7–11 years Ethnicity: Asian Overweight [61]: *n* = 11 Normal weight: *n* = 32	To examine the variations in the gut microbiota between normal weight and overweight and determine the associations between dietary intakes and gut microbiota	16S rRNA gene sequencing	↓ *Bifidobacterium, Turicibacter* and *Clostridiaceae* ↑ *Erysipelotrichaceae UCG-003*, *Lachnospira* and *Peptostreptococcaceae* ≠ Alpha and beta bacterial diversity
Da Silva et al., 2020 [55]	Trinidad	*n* = 51 Age: 6–14 years Ethnicity: Mixed, Africans and Indians Overweight/obese [61]: *n* = 21; BMI > 85th Normal weight: *n* = 30	To describe the composition of the gut microbiota in children with obesity/overweight and children of Normal weight and identify possible associations	16S rRNA gene sequencing	↓ *Bifidobacterium* and *Bifidobacteriaceae* ↓ alpha diversity ↑ *Firmicutes* and *Lactobacillus*
Chen et al., 2020 [16]	China	*n* = 51 Age: 6–11 years Ethnicity: Asian Obese [62]: *n* = 28 Normal weight: *n* = 23	To evaluate gut microbial biodiversity between obese and normal weight children	16S rRNA gene sequencing	↓ *Bacteroidetes*, *Oscillospira* and *Dialister* ↓ reduced alpha diversity and observed species ↑ *Faecalibacterium*, *Phascolarctobacterium*, *Lachnospira*, *Megamonas*, and *Haemophilus*
Shin et al., 2020 [42]	Korea	*n* = 46 Age: 5–13 years Ethnicity: Asian Obese [63]: *n* = 22 Normal weight: *n* = 24	To compare the gut microbiota composition between obese Korean and normal weight children	16S rRNA gene sequencing	↓ *Bacteroidetes*, *Bacteroides ovatus, Porphyromonadaceae*, *Rikenellaceae*, *Bacteroidaceae, Devosia_f*, *Leptotrichiaceae*, *Odoribacteracea* and *Staphylococcaceaeat* ↑ *Actinomyces*, *GL872355_g*, *Lachnospiraceae, Weissella* and *Romboutsia*
López-Contreras et al., 2018 [48]	Mexico	*n* = 138 Age: 6–12 years Ethnicity: Latino Obese [36]: *n* = 71 Normal weight: *n* = 67;	To analyze the gut microbiota composition between obese and normal weight children	16S rRNA gene sequencing	↓ unclassified *Christensenellaceae* ↑ *Bacteroides eggerthii*
Mbakwa et al., 2018 [51]	Netherlands	*n* = 295 Age: 6–7 years Ethnicity: Caucasian Overweight/obese [64]: *n* = 27 Normal weight: *n* = 268	To examine the composition of the gut microbiota of school-aged children in association with weight	16S rRNA gene sequencing	↓ *Akkermansia*, *Sutterella wadsworthensis, Burkholderia*, and *Marvinbryantia formatexigens* ↑ *Streptococcus bovis*
Méndez-Salazar et al., 2018 [49]	Mexico	*n* = 36 Age: 9–11 years Ethnicity: Latino Obese [61]: *n* = 12 Normal weight: *n* = 24	To compare bacterial richness and diversity of the gut microbiota in Mexican children according to weight categories	16S rRNA gene sequencing	↓ Bacterial richness and diversity ↓ *Bacteroidetes* ↑ *Proteobacteria* and *Bilophila phylum*
Gao et al., 2018 [43]	China	*n* = 77 Age: obese 6.8 ± 1.6 years; control 6.0 ± 2.7 years Ethnicity: Asian Obese [61]: *n* = 39; Age = 6.8 ± 1.6 Normal weight: *n* = 38; Age = 6.0 ± 2.7	To analyse the differences in the structure of intestinal flora between obese and normal weight children	16S rRNA gene sequencing	↓ Diversity ↑ *Bacteroidetes phylum* ↓ *Candidatus*, *Actinobacteria*, *Firmicutes*, and *Verrucomicrobia phylum* ↑ *A. histaminiformans*, *B. plebeius*, *B. dorei*, *B. wadsworthia*, *C. symbiosum*, *M. funiformis*, *P. distasonis*, *P. excrementihominis*, *P. stercorea*, and *O. formigenes*
Riva et al., 2017 [52]	Italy	*n* = 78 Age: 6–16 years Ethnicity: Caucasian Obese [61]: *n* = 42 Normal weight: n = 36	To describe the gut microbiota composition in children with and without obesity	16S rRNA gene sequencing	↓ *Bacteroidetes* and *Bacteroidaceae* ↑ *Firmicutes* and *Ruminococcaceae*
Hou et al., 2017 [44]	China	*n* = 143 Age: 3–18 years Ethnicity: Asian Obese: *n* = 87 Normal weight: *n* = 56	To examine differences in gut microbiota between obese and healthy children	16S rRNA gene sequencing	↓ Gram-negative bacteria: *Verrucomicrobia* and *Lentisphaerae* ↑ F/B ratio ↑ *Firmicutes*, *Enterococcus* and *Blautia* ↑ *Proteobacteria*, *Sutterella* and *Klebsiella* ↑ *Actinobacteria* and *Collinsella*
Borgo et al., 2017 [53]	Italy	*n* = 61 Age: 10.03–0.68 years Ethnicity: Caucasian Obese [61]: *n* = 28 Normal weight: *n* = 33	To assess the biodiversity of gut microbiota in obese and non-obese children	16S rRNA gene sequencing	↓ *Akkermansia muciniphyla* ↓*Bacteroides*/*Prevotella* ↓ *Candida* spp., *F. prausnitzii*, and *Saccharomyces* spp.
Ignacio et al., 2016 [50]	Brazil	*n* = 84 Age: 3–11 years Ethnicity: Latino Overweight/Obese [61]: *n* = 54 Normal weight: *n* = 30	Analyse faecal samples for bacteria composition and sought a correlation between the body mass index and these bacteria	16S rRNA gene sequencing	↑ *B. fragilis*, *Lactobacillus* spp. ↓ *Bifidobacterium* spp.
Gao et al., 2015 [45]	China	*n* = 126 Age: obese 6.8 ± 2.1 years; control 6.8 ± 2.4 years Ethnicity: Asian Obese [61]: *n* = 64 Normal weight: *n* = 62	To investigate the correlation between obesity and imbalance of gut microbes in children	16S rRNA-based qPCR	↓ *Bifidobacteria* ↓ B/E ratio ↑ *E. coli*
Bervoets et al., 2013 [54]	Belgium	*n* = 53 Age: 6–16 years Ethnicity: Caucasian Obese [65]: *n* = 26 Normal weight: *n* = 27	To examine the composition of the gut microbiota composition in obese and non-obese children	16S rRNA gene sequencing	↓ *B. vulgatus* ↑ F/B ratio ↑ *Lactobacillus* spp.
Xu et al., 2012 [46]	China	*n* = 175 Age: 7–13 years Ethnicity: Asian Overweight/obese [62]: *n* = 84 Normal weight: *n* = 91	To explore correlations between the composition of the gut microbiota and obesity in children from Kazakh.	16S rRNA gene sequencing	↓ *Bacteroidetes* ↓ F/B ratio

*Abbreviations: BMI*, body mass index; *rRNA*, ribosomal ribonucleic acid; *SD*, standard deviation; *qPCR*, real-time polymerase chain reaction; *F/B ration*, ratio of *Firmicutes* and *Bacteroidetes* spp. species; *B/E ratio*, ratio of *Bifidobacteria* and *E. coli*; *B/F ratio*, Bacteroidetes to *Firmicutes* ratio. Symbols: ≠, no changed or no difference; ↑, increased; ↓, decreased.

**Table 2:** Gut microbiota variations in children with overweight and obesity before and after physical activity interventions.

Reference	Country	Sample	Aim	Methodology	Baseline Analysis	Post Intervention Analysis
Morán-Ramos et al., 2022 [56]	Mexico	Obese [36] male children aged 11–14 years old (*n* = 6)	To analyze the composition of the gut microbiota in obese Mexican children before and after a 6-week intervention	6-week multidimensional intervention. Hypo-energetic diet and moderate to intense physical activity; the recommendation is to achieve 150 min per week. Faecal samples before and after the intervention. 16S rRNA gene sequencing	*Bacteroidetes* (46.5%), *Firmicutes* (45.7%), and *Proteobacteria* (3.7%)	↑ *Odoribacter* (*Bacteroidetes*) ≠ Gut microbiota composition ≠ Gut microbiota diversity Significant proportion of gut microbial variation (individual divergence)
Cho 2021 [19]	Korea	Children (*n* = 60): Obese children [63] (*n* = 36) Fat loss group aged 10.0 ± 2.4 (*n* = 17) Fat gain group aged 10.3 ± 2.7 (*n* = 19) Normal weight children aged 8.1 ± 1.5 years old (*n* = 24)	To evaluate variations in the gut microbiota after an 8-week program	8-week weight reduction program (individual nutritional counselling and physical activity) Questionnaires on general lifestyle Faecal samples before and after a 2-month 16S rRNA gene sequencing	Dysbiotic features of obese children compared with control group: ↑ *Blautia* ↑ *Dorea* ↑ *E. hallii* ↑ *Fusicatenibacter* ↓ Bacteroidetes ↓ *Oscillibacter* ↓ *Parabacteroides*	After intervention, fat loss group showed: ↑ *Firmicutes* ↑ *Clostridiales* orde r↓ Bacteroidetes phylum ↓ *Bacteroides* genus ↓ Microbial richness ≠ beta diversity
Huang et al., 2020 [57]	China	Obese [62] adolescents aged 9–16 years old (*n* = 24)	To investigate the impact of an exercise program and dietary restrictions over 6 weeks on gut microbiome and central hemodynamics in obese adolescents	6-week program Endurance and strength exercises, 5 h per day, 6 days a week Calorie-restricted diet Faecal samples before and 24 h after the last session 16S rRNA gene sequencing	↑ *Lactobacillales*, *Bacilli*, *Streptococcaceae*, *Streptococcus*, and *Veillonellawere* (members of *Firmicutes phylum*)	↓ *Firmicutes* ↑ *Bacteroidetes* ↓ F/B ratio ↑ Alpha diversity ↑ *Lentisphaeria* ↓ *Lactobacillales*, *Bacilli*, *Streptococcaceae* and *Veillonella* (members of *Firmicutes phylum*)
Quiroga et al., 2020 [23]	Spain	Children aged 7–12 years old (*n* = 53): Obese group (O, *n* = 39) Healthy control group (Hc, *n* = 14) Obese group (O): Training (Oe, *n* = 25) Control (Oc, *n* = 14)	To study the influence of a 12-week training program on gut microbiota and inflammation in children with obesity	12-week program Strength and endurance training (2 times per week) Faecal samples before and after the program 16S rRNA gene sequencing	Obese group: ↑ *Bacteroidetes* ↑ *Proteobacteria* ↓ *Firmicutes* ↓ *Actinobacteria*	After intervention, the Oe group showed: ↑ *Firmicutes phylum* (↑ beneficial bacterial genera: *Blautia*, *Dialister*, and *Roseburia*) ↓ *Proteobacteria phylum* (↓ *Gammaproteobacteria* class)
Bai et al., 2018 [59]	USA	Children aged 7–18 years old (*n* = 267) 62.9%—normal BMI level 21.0%—underweight 16.1%—overweight and obese [36]	To investigate the relationships between gut microbiota, body mass index and lifestyles (exercise and diet)	Retrospective questionnaire about dietary habits and exercise frequency 16S rRNA gene sequencing	Overall analysis: ↑ *Firmicutes* ↑ *Bacteroidetes* ↑ *Proteobacteria*	Children who Daily Exercise: ↑ *Firmicutes phylum* ↑ Alpha-diversity ↑ *Clostridiales* ↑ *Lachnospiraceae* ↑ *Erysipelotrichaceae*
Santacruz et al., 2009 [58]	Spain	Overweight [65] adolescents aged 13–15 years (*n* = 36): High weight–loss (>4.0 kg of weight loss, *n* = 23) Low weight–loss (<2.0 kg of weight loss, *n* = 13)	To evaluate the effect of a weight reduction program on the body weight and gut microbiota in overweight adolescents.	10-week with hypocaloric diet (10 to 40%) and physical activity (15 to 23 kcal/kg body weight/week). Diary intake records Faecal samples before and after the program Quantitative real-time PCR	High weight–loss group: ↑ *B. fragilis* ↑ *C. leptum* ↓ *C. coccoides* ↓ *Lactobacillus* ↓ *Bifidobacterium*	Both groups: ↓ *Clostridium coccoides* ↓ *B. longum* ↓ *B. adolescentes* ↑ *Bacteroides fragilis* ↑ *Lactobacillus* High weight–loss group: ↓ *C. coccoides* ↓ *B. longum* ↑ *Bacteroides fragilis* ↑ *Lactobacillus* High weight–loss vs. Low weight–loss: ↓ *C. coccoides* ↓ *Lactobacillus* ↓ *Bifidobacterium (B. breve and B. bifidum)* ↑ *B. fragilis* ↑ *C. leptum* ↑ *B. catenulatum*

*BMI* body mass index; *lb* pound; *rRNA* ribosomal ribonucleic acid; *OTUs* operational taxonomic units; *kg* kilogram; *h* hours; *OB* obese; *NW* normal weight; *qPCR* real-time polymerase chain reaction; *F/B* ratio of *Firmicutes* and *Bacteroidetes*. Symbols: ≠, no change or no difference; ↑, increased; ↓, decreased.

## Data Availability

The original contributions presented in the study are included in the article. Further inquiries can be directed to the corresponding authors.
